# Once induced, it lasts for a long time: the structural and molecular signatures associated with depressive-like behavior after neonatal immune activation

**DOI:** 10.3389/fncel.2022.1066794

**Published:** 2022-12-22

**Authors:** Julia N. Khantakova, Natalia P. Bondar, Elena V. Antontseva, Vasiliy V. Reshetnikov

**Affiliations:** ^1^Institute of Cytology and Genetics, Siberian Branch of Russian Academy of Sciences (SB RAS), Novosibirsk, Russia; ^2^Federal Government-Funded Scientific Institution Research Institute of Fundamental and Clinical Immunology (RIFCI), Novosibirsk, Russia; ^3^Department of Natural Sciences, Novosibirsk State University, Novosibirsk, Russia; ^4^Department of Biotechnology, Sirius University of Science and Technology, Sochi, Russia

**Keywords:** depression, neuroinflammation, microglia, astrocytes, HPA, oxidative stress, cytokines

## Abstract

Adverse factors such as stress or inflammation in the neonatal period can affect the development of certain brain structures and have negative delayed effects throughout the lifespan of an individual, by reducing cognitive abilities and increasing the risk of psychopathologies. One possible reason for these delayed effects is the neuroinflammation caused by neonatal immune activation (NIA). Neuroinflammation can lead to disturbances of neurotransmission and to reprogramming of astroglial and microglial brain cells; when combined, the two problems can cause changes in the cytoarchitecture of individual regions of the brain. In addition, neuroinflammation may affect the hypothalamic–pituitary–adrenal (HPA) axis and processes of oxidative stress, thereby resulting in higher stress reactivity. In our review, we tried to answer the questions of whether depressive-like behavior develops after NIA in rodents and what the molecular mechanisms associated with these disorders are. Most studies indicate that NIA does not induce depressive-like behavior in a steady state. Nonetheless, adult males (but not females or adolescents of both sexes) with experience of NIA exhibit marked depressive-like behavior when exposed to aversive conditions. Analyses of molecular changes have shown that NIA leads to an increase in the amount of activated microglia and astroglia in the frontal cortex and hippocampus, an increase in oxidative-stress parameters, a change in stress reactivity of the HPA axis, and an imbalance of cytokines in various regions of the brain, but not in blood plasma, thus confirming the local nature of the inflammation. Therefore, NIA causes depressive-like behavior in adult males under aversive testing conditions, which are accompanied by local inflammation and have sex- and age-specific effects.

## Highlights

- Neonatal inflammation leads to the development of depression-like behavior only under aversive conditions in adult males.
- Males exhibiting depressed behavior show long-term elevated activation of microglia and astrocytes, which is accompanied by high oxidative stress and high reactivity of the HPA axis.
- The consequences of neonatal inflammation are sex-specific: more pronounced in males than females.
- In males after neonatal immune activation (NIA), there are signs of only local inflammation in brain tissues.

## Introduction

Depression is a general mental disorder that can be either a syndrome of another disease or an independent illness: major depressive disorder (MDD). According to the WHO, >7% of adults have a diagnosis of depression, and people at ages 12–25 have the highest prevalence rates according to data from the National Institute of Mental Health (NIMH; Center for Behavioral Health Statistics, 2021)^1^. The population of patients with depressive behavior is heterogeneous with respect to symptoms, treatment responses, and biological correlates. The pathogenesis of depression includes genetic predisposition, disturbances in the synthesis and activities of monoamines in the central nervous system (CNS), aberrations in the functioning of the hypothalamic–pituitary–adrenal (HPA) axis, alterations in brain regions/functions, and long-term low-grade neuroinflammation (Beijers et al., 2019).

More than 30 years ago, it was demonstrated that inflammation plays a substantial role in the pathogenesis of depression (Maes et al., 1990), especially its treatment-resistant subtype (Dowlati et al., 2010). Several “inflammatory” models of depression have been devised, such as immune activation during pregnancy (Patterson, 2011; Bilbo et al., 2018) or during the early postnatal period (Lucchina et al., 2010; Liang et al., 2019; Cao et al., 2021) or administration of proinflammatory cytokines (Udina et al., 2012); these models have revealed an effect of immune-system activation on the pathogenesis of neuropsychiatric disorders. Moreover, even noninfectious stress activates the immune system and triggers “sterile” inflammation (Iwata et al., 2013) associated with changes in neurotransmitter and neuropeptide systems. Various meta-analyses have proven that in approximately a third of patients with depression, there are biological signs of peripheral inflammation that disappear during an antidepressant treatment, e.g., psychotherapy. Among inflammatory markers, elevated levels of C-reactive protein, IL-6, and TNF are the most reliable markers of depression (Haapakoski et al., 2015; Enache et al., 2019). The latest trial showed that people with recurrent, especially treatment-resistant depression, also feature an elevated level of IL-17, which can become an additional diagnostic marker of treatment-resistant depression (Gałecka et al., 2021; Nothdurfter et al., 2021). Clinical trials suggest that when the inflammation level is high initially (C-reactive protein >5 mg/dL), combined use of cytokine antagonists (e.g., TNF antagonist infliximab) and antidepressant therapy can improve the course of depression (Raison et al., 2013).

Newborns are very susceptible to various types of infections owing to the continued maturation/formation of various organs and systems of organs, including the immune system (Yu et al., 2018). A specific feature of the immune system of newborns is the immature status of adaptive immune responses. Therefore, innate immune responses are crucial for survival in the early neonatal period (Yu et al., 2018). It is known that some biomolecules (cytokines, adhesion molecules, and the complement system) participating in innate immunity are expressed in the healthy brain and play an important part in neurogenesis, neuronal migration and differentiation, synapse formation, and neuroplasticity (Irwin and Cole, 2011). Accordingly, either deficiency or overactivation of the immune system early in life can potentially have long-term detrimental effects on brain development and behavior. One of the possible reasons is persisting low-grade inflammation in the body and brain, which in stressful situations in adulthood can have the same impact as direct activation of the immune system. In longitudinal studies, perinatal infection is reported to raise the risk of mood disorders, including anxiety and MDD (Chu et al., 2019; Toenders et al., 2021). In a large-scale study called the Avon Longitudinal Study of Parents and Children (ALSPAC), researchers found that early-childhood infections correlate with the development of depressive symptoms in mid-adolescence (Chaplin et al., 2020). Children with elevated levels of IL-6 and C-reactive protein at 9 years of age are at a higher risk of depression onset by 18 years of age (Khandaker et al., 2018). Epstein–Barr virus exposure at age 4 years is associated with subsequent risk of definite psychotic experiences in adolescence (Khandaker et al., 2014).

Investigation into rodent models confirms that inflammatory processes during the embryonic and neonatal period can result in the development of neuropsychiatric disorders later in life (Meyer et al., 2009; Liang et al., 2019; Cao et al., 2021; Khantakova et al., 2022a). The mechanism of neuronal anomalies during maternal immune activation involves impairments of active development of brain structures (Gałecka et al., 2021). On the other hand, molecular mechanisms of the neurobehavioral disorders that emerge after neonatal immune activation (NIA) are not yet fully understood. In 2012, in their review, Bilbo and Schwarz comprehensively examined the delayed impact of perinatal infection on immune responses and cognition later in life (Bilbo and Schwarz, 2012). Those authors distinguished the direct and indirect effects of immune activation on neuronal functions: (1) the direct influence of immune activation is the introduction of irreversible alterations or disturbances into certain neural processes, e.g., neurotransmission and synaptic plasticity, which is key to learning and memory; and (2) by the indirect effect, those authors mean a reprogramming of the immune function of the adult human body, i.e., a change in how a response to a subsequent immune challenge takes place in adulthood (Bilbo and Schwarz, 2012). Indeed, there is growing evidence of relations among low-grade inflammation, levels of proinflammatory cytokines, and changes in neurotransmitter and neuroendocrine functioning in psychiatric disorders, during depressive and anxious behavior, and in MDD (Chu et al., 2019; Toenders et al., 2021).

One of the simple experimental approaches to the research on the influence of the immune system on the developing brain is to induce antibacterial immune responses without direct administration of bacterial pathogens, i.e., *via* injection of bacterial endotoxin: lipopolysaccharide (LPS). The use of the mimetic rather than a live bacterium has a number of advantages: biological safety, dose-dependent intensity of the immune response (Furube et al., 2018), and the ability to quantify cytokines (Yan and Kentner, 2017; Custódio et al., 2018; Berkiks et al., 2019a) and the expression of immune-system–related genes (Cao et al., 2021; Kvichansky et al., 2021) in the blood and various tissues, including the brain. Additionally, the effect of mimetic administration is time-limited: 24–48 h depending on the dose (Furube et al., 2018). These advantages allow us to set the exact time of immune activation corresponding to specific periods of neuroimmune-axis development; this approach is especially relevant for studies on the influence of an immune challenge on brain dysfunction (Liang et al., 2019; Luo et al., 2021; Zhao et al., 2022). It is worth noting that mimetics cannot simulate the full range of immune responses caused by a bacterial infection; they mimic only cytokine-related responses of the acute phase of inflammation (Schwarz and Bilbo, 2011).

Overall, it is now known that immune activation at an early age can lead to impaired brain development and may influence behavior later in life. Nevertheless, the limitations of human studies and the heterogeneity of animal models make it difficult to pinpoint the impact of neonatal inflammation on the risk of depression at an older age. To address this issue, here we analyze the results of animal studies involving a model of LPS-induced neonatal inflammation in rodents (mice and rats). Besides, we discuss long-term molecular changes in brain structures and their relations with depressive-like behavior in adolescents and adults.

## The Search Strategy

Preferred Reporting Items for Systematic Reviews and Meta-Analyses (PRISMA) guidelines were followed during the preparation of this review. Two authors (JK and EA) searched Scopus databases independently to select the articles that would best reflect the assumptions of the study in the “Introduction” Section. “Neonatal immune activation” was combined with the term “depression, ” “forced swim, ” “tail suspension, ” “anhedonia, ” and “rodent” to find relevant articles (title/abstract/key). The present authors included only original articles in the English language that were related directly to LPS in NIA. The databases were searched in July–August 2022. The authors retrieved 239 records from Scopus (Supplementary Material) and excluded articles having the year of publication before 1990.

After an initial review, 30 articles were excluded because they were review articles, editorials, commentaries, letters to the editor, or not full-length article. Next, 154 articles, involving maternal/prenatal/*in utero* immune activation, administration of a mimetic after postnatal day 14, early-life social stress (separation), ischemia, alcohol or other mimetics, or cell culture research were excluded from the study. Eight studies on other types of behavior (e.g., pups’ behavior, anxiety, memory, learning, and locomotor activity) as well as studies on a long-term molecular change without an analysis of behavior were also excluded from the analysis (Supplementary Material). Thus, we selected 25 full-length animal research articles for the analysis, all related to the development of depressive-like behavior in adolescents (postnatal days 30–45) or adult rodents (after postnatal day 60) after neonatal administration of LPS and associated molecular patterns. In three studies, their authors investigated long-term alterations in both age groups.

The consequences of NIA in adolescents are studied in eight research articles, whereas in adults, in 20 studies, among which, three articles (Dinel et al., 2014; Tishkina et al., 2016; Custódio et al., 2018) address long-term alterations in both age groups of animals. In 10 (40%) studies, experiments are conducted on different strains of mice, and in 15 (60%) studies, on different strains of rats. In eight (32%) studies, investigators use only males, in 13 (52%) studies, males and females are analyzed separately, and in four (16%) studies, there are mixed groups. In 40% of our selected studies (Table 1), researchers employ the “dual-exposure-to-endotoxin” model of mimetic administration, where different doses of endotoxin (0.025–0.05 mg/kg) are administered twice: on postnatal days 3 and 5. In eight (32%) studies, an endotoxin (0.05–0.1 mg/kg) is administered once on postnatal day 14, and in one study (4%) twice on postnatal day 14–15, already at the end of the stress-hyporesponsive period. Furthermore, in six (24%) studies, 0.025–2.0 mg/kg LPS is administered at various time points during the first 2 postnatal weeks. Nonetheless, regardless of the administration scheme, the administration of LPS drives the activation of microglia and astrocytes (Zhao et al., 2022), upregulation of mRNA expression of *Il-6*, *Il-1β*, and *Tnf* in various brain regions, and of respective protein levels in blood plasma (Dinel et al., 2014; Cao et al., 2021; Zhao et al., 2022), and enhanced production of corticosterone (Lucchina et al., 2010) during the first 24 h. To make the data compatible among the studies, in articles not specifying the sex of the studied animals, we assumed that investigators analyzed a mixed group of animals. To calculate the percentage of animals in which certain changes were observed, we utilized data on the number of animals presented in the original articles. In the absence of data on the numbers of females and males in mixed groups, we attributed the reported number of animals to both sexes.

**Table 1:** Characteristics of the 25 original articles included in the analysis.

**Day of analysis**	**Day injection**	**Dose LPS, mg/kg**	**Model**	**Sex**	**Sucrose test**	**FST**	**TST**	**The studied molecular patterns**	**References**
**Adolescent**									
30 d	14	IP, 0.1	CD1 mice	m		immobility = (*n* = 20)		Cytokines (*n* = 20)	Dinel et al. (2014)
30–35 d	3+5	SC, 0.05	Wistar rats	m		immobility = (*n* = 9)		CORT (*n* = 9)	Tishkina et al. (2016)
31 d	14	IP, 0.05	C57Bl/6	m, f	preferences ↓, m (*n* = 5–6) preferences = , f (*n* = 8)	immobility ↑, m (*n* = 5–6), f (*n* = 8)	immobility ↑, m (*n* = 5–6) immobility =, f (*n* = 8)	Cytokines (*n* = 5–6) Microglia (*n* = 5–6) neuronal activity (*n* = 5–6)	Cao et al. (2021)
32 d	14+15	IP, 0.1	Sprague-Dawley rats	n.d.	preferences = (*n* = 7)	immobility = (*n* = 7)		NLRP3 microglia	Lei et al. (2017)
35 d	5+7	IP, 0.05	Swiss mice	m, f	preferences = (*n* = 6–9)	immobility ↑, m immobility =, f (*n* = 7–10)		Cytokines (*n* = 6–8) Oxidative stress (*n* = 6–8)	Custódio et al. (2018)
40–42 d	3+5	IP, 0.05	Sprague-Dawley rats	m, f	preferences = (*n* = 7–8)			Cytokines (*n* = 7–8) CORT (*n* = 7–8)	Yan and Kentner (2017)
40–46	3+5	IP, 0.05	Wistar rats	m		immobility (*n* = 12) N			Rico et al. (2010)
42–48 d	3+5	IP, 0.05	C57Bl/6	m, f		immobility ↑ (*n* = 6–9)	immobility ↑ (*n* = 6–9)	Cytokines (*n* = 3–4) Microglia (*n* = 4) Astrocytes (*n* = 4) Oxidative stress (*n* = 4)	Zhao et al. (2022)
**Adult**									
60 d	3	IP, 0.025	F1 C57Bl/6xBal F1 BalbxC57Bl/6	m, f		C57Bl/6xBalb immobility = ; m (*n* = 10), f (*n* = 15) BalbxC57Bl/6 immobility = ; m (*n* = 10), f (*n* = 14)	C57Bl/6xBalb immobility = ; m (*n* = 10), f (*n* = 15) BalbxC57Bl/6 immobility = ; m (*n* = 10), f (*n* = 14)	CORT (*n* = 3–12)	Lucchina et al. (2010)
60 d	3	IP, 0.05	F1 C57Bl/6xBalb F1 BalbxC57Bl/6	m, f.		C57Bl/6xBalb immobility = ; m (*n* = 6), f (*n* = 8) BalbxC57Bl/6 immobility = ; m (*n* = 10), f (*n* = 8)	C57Bl/6xBalb immobility ↑; m (*n* = 6), f (*n* = 8) BalbxC57Bl/6 immobility = ; m (*n* = 10), f (*n* = 8)	CORT (*n* = 3–12)	Lucchina et al. (2010)
60 d	2+3	SC, 0.025	C57Bl6	m		immobility ↑ (*n* = 20)			Comim et al. (2016)
8–10 wk	14	IP, 0.1	SD rats	m		Immobility = (*n* = 8)			Spencer et al. (2005)
60–70 d	2	IP, 0.05	Wistar rats	m, f		Immobility# ↓ m immobility# = f (*n* = 13)		Cytokines (*n* = 4–6) CORT (*n* = 6)	Bernardi et al. (2014)
70 d	5+7	IP, 0.05	Swiss mice	m, f	preferences = (*n* = 6–9)	immobility ↑, m immobility = , f (*n* = 7–10)		Cytokines (*n* = 6–8) Oxidative stress (*n* = 6–8)	Custódio et al. (2018)
80–90 d	14	IP, 0.1	Sprague-Dawley rats	m, f	preferences = m = f (*n* = 5–7) preferences=* m = f			Cytokines (*n* = 5–6) Oxidative stress (*n* = 5–6)	Kentner et al. (2010)
12 wk	3+5	IP, 0.05	C57Bl/6 × DBA-2	m, f		immobility ↓, m immobility = , f (*n* = 13)		Neurogenesis (*n* = 3–6)	Luo et al. (2021)
12–13 wk	14	IP, 0.25	Wistar rats	m		immobility ↑ (*n* = 5)		Cytokines (*n* = 5) Oxidative stress (*n* = 5) Microglia (*n* = 7) Astrocytes (*n* = 7)	Berkiks et al. (2018a)
90 d	3+5	SC, 0.05	NMRI mice	m, f		immobility ↑ m = f (*n* = 8)	immobility ↑ m = f (*n* = 8)	CORT (*n* = 8)	Doosti et al. (2013)
90 d	14	IP, 0.1	CD1 mice	m		immobility ↑ m (*n* = 20)		Cytokines CORT Neurogenesis (*n* = 8)	Dinel et al. (2014)
90 d	3+5	SC, 0.05	NMRI mice	m	preferences ↓ (*n* = 9)	immobility ↑ (*n* = 9)	immobility ↑ (*n* = 9)	Cytokines (*n* = 9) CORT (*n* = 9)	Majidi et al. (2016)
90 d	3+5	SC, 0.05	C57Bl/6	m, f		immobility ↑ (*n* = 14)	immobility ↑ (*n* = 14)	Microglia (*n* = 8) Astrocytes (*n* = 8) Neurogenesis (*n* = 8)	Liang et al. (2019)
90–97 d	1	IP, 0.25	Wistar rats	m, f		immobility ↑, m immobility = , f (*n* = 12)		Oxidative stress (*n* = 12)	Benmhammed et al. (2019)
96–97 d	3+5	0.05	Long-Evans rats	m, f	preferences = , m (*n* = 15), f (*n* = 15)				Tenk et al. (2013)
3 mth	9	IP, 0.25	Wistar rats	m		immobility ↑ (*n* = 7)		Cytokines Microglia Oxidative stress	Berkiks et al. (2018b)
3 mth	14	IP, 0.25	Wistar rats	m, f		immobility ↑, m immobility = , f (*n* = 8)		Cytokines (*n* = 5) Microglia (*n* = 4) Astrocytes (*n* = 4)	Berkiks et al. (2019b)
103–107 d	3+5	SC, 0.05	Wistar rats	m, f		immobility ↑, m immobility = , f (n = n.d.)		Cytokines (n = n.d.) Microglia (n = n.d.)	Kvichansky et al. (2021)
107 d	3+5	SC, 0.05	Wistar rats	m		immobility ↑ m (*n* = 12)		CORT (*n* = 12)	Tishkina et al. (2016)
120–150 d	14	IP, 0.5	Sprague-Dawley rats	m, f		floating time = , m (*n* = 7–8), f (*n* = 10)			Saavedra et al. (2021)
11 mth	14	IP, 0.25	Wistar rats	m, f		immobility = , m immobility ↑, f (*n* = 8)		Cytokines (*n* = 4) Oxidative stress (*n* = 4) Microglia	Berkiks et al. (2019a)

Comments: the studies in the table are arranged according to the age of animals during behavioral testing. The number of animals per group (n) in a given study is shown in parentheses. The presented data were retrieved from original articles and examined. A detailed description of molecular changes is given in our article below in the relevant tables. Abbreviations: IP, intraperitoneal; SC, subcutaneous; m, male; f, female; d, day; mth, month; wk, week; n.d., no data; PND, postnatal day; ↑/↓, an increase or decrease in a parameter compared with a control, respectively; = , no difference from the control; CORT, corticosterone; FST, forced swim test; TST, tail suspension test; n, the number of animals per group; ^#^after an adult LPS rechallenge; *25–30 min after aversive conditions (the forced swim test, tail suspension test, or restraint test).

## Assessment of Depressive-Like Behavior After NIA

To quantify depressive-like behavior in animal models, researchers commonly use the tail suspension test (Steru et al., 1985), the forced swim test (Porsolt et al., 1977), and the sucrose or saccharine preference test (Willner et al., 1987). Less commonly, the open field test, the novelty-suppressed feeding test (Dulawa and Hen, 2005), and the elevated plus maze test are employed, which primarily evaluate the general locomotor activity and anxiety-like behavior rather than depressive behavior; for this reason, studies involving such tests are not analyzed in this review.

The forced swim test is the most common assay of depressive behavior. In the forced swim test, an animal is placed in an aversive environment: a container of water from which the animal cannot escape. After unsuccessful attempts to get out, the animal stops struggling (and floats with minimal movements needed only to keep the nose above the water). The length of time of the animals’ trying to find a way out under the unavoidable aversive conditions reflects the degree of their behavioral despair and correlates well with the therapeutic action of antidepressants (Kara et al., 2018). The tail suspension test is similar to the forced swim test and also assesses the degree of behavioral despair but does not involve water immersion, which is a strong stressor and can induce hypothermia in animals. In the tail suspension test, rodents are hung by the tail, and the length of time is also determined after which the animals stop looking for a way out of the aversive situation and assume an immobile posture. The sucrose or saccharine preference test is used to evaluate the ability to experience pleasure (Willner et al., 1987). One of the symptoms of depression is anhedonia, i.e., the inability to experience pleasure. A decrease in the consumption of sucrose, which rodents normally prefer, indicates the development of anhedonia.

Analysis of the literature indicates that NIA does not lead to anhedonia. Only one of four studies on sucrose preference in both adolescent and adult rodents shows a reduction in sucrose intake (Table 1; Majidi et al., 2016; Cao et al., 2021). This indicates the absence of symptoms of depressive-like behavior in a steady state. By contrast, placement of animals with a history of NIA into aversive settings (the forced swim test or tail suspension test) leads to the manifestation of pronounced symptoms of depressive-like behavior in adult animals, as reported in 14 out of 20 studies (Table 1). Of note, males present with depressive behavior more frequently (55%–60% of animals analyzed) than females do (23%–32% of animals analyzed), implying sex-specific effects of NIA. In one work, on the contrary, a decrease in depressive behavior in adult males is demonstrated, possibly due to low sensitivity of F1 C57Bl/6 × DBA-2 hybrids to the forced swim test (Luo et al., 2021). Unexpectedly, an increase in immobility in the forced swim test or tail suspension test was observed only in three out of seven studies on adolescent rodents (postnatal days 30–48), and there are no pronounced differences between females (21%–26% of the studied animals according to data from the forced swim test) and males (27%–34% of the studied animals according to data from the forced swim test). Thus, neonatal inflammation in most cases leads to depressive-like behavior in adult males, but not in females or adolescents of both sexes, and only under aversive testing conditions.

Of note, under stable environmental conditions in both humans and rodents, it is difficult to analyze delayed behavioral symptoms after neonatal inflammation. For this reason, investigators often use various aversive conditions to assess depressive behavior and associated molecular aberrations. As aversive conditions, researchers have utilized the forced swim test or tail suspension test (Doosti et al., 2013; Berkiks et al., 2018a; Custódio et al., 2018; Kvichansky et al., 2021) or the restraint test (Majidi et al., 2016), which may reflect an effect of acute stress. Or, in the case of acute inflammatory stress, the administration of various doses of LPS to adult animals has been used (Kentner et al., 2010; Lucchina et al., 2010; Bernardi et al., 2014; Yan and Kentner, 2017). Surprisingly, in one study, after an adult LPS rechallenge, researchers documented a decrease, not an increase, in the depressive behavior of males during the forced swim test (Bernardi et al., 2014). Those authors explain this result by the induction of endotoxin tolerance in response to neonatal LPS exposure. The absence of other studies with such a design does not allow us to either confirm or refute the validity of this conclusion.

The lack of an effect of neonatal inflammation in female rodents may be due to increased variation of outcomes owing to the influence of the estrous-cycle stage on behavior. Only one of the presented studies addresses the influence of this cycle’s stage on the behavior of females (one of 17 studies for all ages, or 6%). To obtain relevant results, it is necessary to conduct more systematic studies on females, increase the sample size, and subdivide the animals into groups according to the stage of the cycle.

## Neuroglial Alterations After NIA Associated with Depression

Thanks to advances in neuroimaging studies, brain regions have been identified that are significantly impaired in patients with MDD. In the brain, they include those areas that regulate motivation, locomotor activity, arousal, and anxiety (Miller and Raison, 2016). Various areas of the frontal cortex and limbic regions are involved in the regulation of these types of behavior, for example, the amygdala, hippocampus, and striatal regions including the caudate, putamen, and nucleus accumbens (Li B.-J. et al., 2018; Zhang et al., 2018). Neuron division in most regions of the brain is already completed by the time of birth but continues in the dentate gyrus of the hippocampus and subventricular zone. Therefore, the main negative effect of NIA is absorbed by cells that perform immunocompetent and homeostatic functions in the brain: microglia and astroglia. These cells actively divide and undergo a series of morphological alterations during the neonatal period (Bilimoria and Stevens, 2015; Abbink et al., 2019). Changes in the morphology and amounts of microglia and astroglia, just as processes of neurogenesis, are dynamic indicators of the state of the brain, and their disturbances point to pathological conditions, including neuroinflammation.

### Microglia

The main immunocompetent cells of the brain are microglia. Due to their cytokine-synthesizing and phagocytic activities, these cells influence the development and maturation of CNS structures (Bilimoria and Stevens, 2015), participate in normal formation and maturation of neural circuits during development, maintain the neuronal cell pool, and mediate normal synaptic pruning, synapse maturation, and connectivity, thereby regulating the number of synapses and receptor expression (Ji et al., 2013; Zhan et al., 2014). Peripheral inflammatory stimuli induce an immunological response in the brain thereby leading to microglia activation (Hoogland et al., 2015), migration, enhancement of their phagocytic properties, synthesis of inflammatory biomolecules, and recruitment of peripheral immune cells to the brain, thus shaping a cerebral inflammatory environment that may strongly affect neuron functions. An excessive and prolonged microglial response can become harmful and cause persistent neuroinflammation and neurodegeneration.

The latest studies confirm that microglial activation in depression-related brain regions such as the prefrontal cortex and anterior cingulate cortex plays a central role during episodes of severe depression (Setiawan et al., 2015). Thanks to positron emission tomography, researchers have been able to detect an increase of activated-microglia-related neuroinflammation in the right hippocampus of bipolar patients (Haarman et al., 2014) and in the anterior cingulate cortex, insula (Setiawan et al., 2015; Holmes et al., 2018), and the prefrontal cortex (Setiawan et al., 2015) of patients during a major depressive episode. As symptoms of depression abate, there is a decline in microglial activity (Li H. et al., 2018). In depressed suicides, in the anterior cingulate cortex and anterior middle cingulate cortex, there is higher activity (Torres-Platas et al., 2014) and density (Steiner et al., 2011) of microglial cells producing quinolinic acid: one of the products of the inflammatory kynurenine pathway of tryptophan metabolism (Myint and Kim, 2014), indicating an enhanced microglial response to cytokine signals. All this confirms the close link between depression and the state of microglial cells.

The relation between microglial activity and the formation of depressive behavior in animals after NIA is reported in seven studies, among which, in three studies, researchers assess changes in adolescents, and in four studies, in adult animals (Table 2). Immunostaining for microglia/macrophage-specific marker Iba-1 is performed to characterize microglia morphology and distribution and to determine the number of microglia (Imai et al., 1996). In adolescents (postnatal days 30–45), after NIA, two out of the three studies show an increase in microglial activity (the number and staining intensity of Iba^+^ cells) in the hippocampus and prefrontal cortex (Lei et al., 2017; Cao et al., 2021). In males with a history of NIA, in the anterior cingulate cortex, in addition to an increase in the number and staining intensity of Iba^+^ cells, there is upregulation of CD68 and MHC II and an increase in microglial phagocytosis of synapses; these signs are associated with microglial activation (Cao et al., 2021). Importantly, only one study points to a link between microglial activation and the development of depressive behavior in adolescence (postnatal days 35–42; Cao et al., 2021). In other studies, on animals of this age, investigators find either microglial activation without the development of depressive behavior (Lei et al., 2017) or depressive behavior not accompanied by microglial activation (Kvichansky et al., 2021).

**Table 2:** Glial alterations and neurogenesis after NIA.

**Time after injection**	**PND**	**Dose LPS, mg/kg**	**Model**	**Sex**	**Depressive behavior**	**Microglia**	**Astrocytes**	**Neurogenesis**	**References**
**Adolescent**									
30 d	3+5	SC, 0.05	Wistar rats	m, f	↑	fractal dimension Iba^+^ = m>f CA1 VH number Iba^+^ = m<f DG DH			Kvichansky et al. (2021)
32 d	14	IP, 0.1	Sprague-Dawley rats	m, f	=	cell count Iba^+^ ↑ DG, PFC			Lei et al. (2017)
35–45 d	14	IP, 0.05	C57Bl/6	m	↑	ACC: Iba^+^ N Iba^+^* (number and intensity)↑ spine density ↓ microglial phagocytosis* ↑		ACC-Glu: NeuN =	Cao et al. (2021)
**Adult**									
12 wk	3+5	IP, 0.05	C57Bl/6xDBA-2	m, f	m ↓ f =			DCX^+^= m, DH DCX^+^ ↑ f, DH DCX^+^ = m, f VH BrdU^+^ = m, f	Luo et al. (2021)
12–13 wk	14	IP, 0.25	Wistar rats	m	↑	percentage of activated microglia Iba^+^ ↑ DG, CA1, CA3	number GFAP^+^ ↑ DG, CA1, CA3		Berkiks et al. (2018a)
90 d	14	IP, 0.1	CD1 mice	m	↑			DCX^+^ = DG DCX^+^ # ↓ DG	Dinel et al. (2014)
60–90 d	3+5	SC, 0.05	C57Bl/6	m, f	↑	intensity Iba = DG	intensity of GFAP = DG	BrdU = BrdU/NeuN ↓ BrdU/DCX ↑	Liang et al. (2019)
3 mth	14	IP, 0.25	Wistar rats	m, f	m ↑ f =	DG: DH, VH number/reactive Iba^+^ ↑ m<f, CA1: DH, VH number/reactive Iba^+^ ↑ m, f CA3: DH, VH number/reactive Iba^+^ ↑ m, f	DH GFAP^+^ = DG m = f GFAP^+^ ↑ CA1, CA3 m<f VH f: GFAP^+^ ↑ DG, CA1, CA3 m: GFAP^+^ = DG, CA1, CA3		Berkiks et al. (2019b)
11 mth	14	IP, 0.25	Wistar rats	m, f	m = f ↑	microglial complexity ↑ f CA1, CA3 microglial complexity ↑ m<f PFC cell body size ↑ f CA1, PFC cell body size ↑ m<f CA3			Berkiks et al. (2019a)

Comments: changes in depressive behavior were assessed if at least one of the special tests showed such behavior (Table 1). Abbreviations: IP, intraperitoneal; SC, subcutaneous; m, male; f, female; d, day; mth, month; wk, week; PND, postnatal day; ↑/↓, an increase or decrease in a parameter compared with a control, respectively; = , no difference from the control; m > f/m vs. f, the comparison of the magnitude of changes between females and males. CA1 and CA3, regions of the hippocampus; DG, dentate gyrus; HC, hippocampus; VH, ventral hippocampus; DH, dorsal hippocampus; PFC, prefrontal cortex; Iba, a marker of microglia; GFAP, glial fibrillary acidic protein, a marker of astrocytes; DCX, doublecortin, a marker of immature neurons; BrdU, bromodeoxyuridine, an indicator of actively dividing cells; NeuN, neuronal nuclei, an indicator of mature neurons; BDNF, brain-derived neurotrophic factor. ^#^after an adult LPS rechallenge; *25–30 min after aversive conditions (the forced swim test, tail suspension test, or restraint test).

In adult animals (after postnatal day 60) with a history of NIA, microglial activation is detectable in three of four males and in two of four females; however, these alterations of microglia, as in adolescents, do not always coincide with behavioral changes. An increase in the activity and complexity of microglia has been shown in the hippocampus (dorsal and ventral) and prefrontal cortex in animals of both sexes, and this active state of microglia persists for up to 11 months (Bernardi et al., 2014; Berkiks et al., 2018a; Berkiks et al., 2019b). Nonetheless, it is worth mentioning that these results are presented in studies by the same group of authors and involving a large dose of LPS (250 μg/kg), whereas at lower doses (50 μg/kg), no increase in activity has been found in microglia after NIA even by the age of 3 months (Liang et al., 2019), despite the formation of depressive behavior in the animals.

Therefore, just as in MDD, after NIA, the activated status of microglia persists for a long time in rodents. Nevertheless, the development of depressive behavior is age-dependent and dose-dependent.

### Astrocytes

Astrocytes are another cell population taking part in brain homeostasis. They exert control over glucose uptake, regulate glutamate homeostasis, release gliotransmitters in response to neural activity, and provide neurotrophic support for neurons (Chung et al., 2015). Besides, they are involved in the interaction of resident and infiltrating cells during neuroinflammation through the production of IL-17 and IFN-γ (Miljkovic et al., 2007; Colombo and Farina, 2016). To identify astrocytes, it is customary to perform immunostaining for glial fibrillary acidic protein (GFAP). It is a cytoskeletal protein with structural and motor functions but is also linked with communication between neurons and astrocytes. GFAP gene activation and protein induction appear to play a critical role in astroglia activation (astrogliosis; Eng and Ghirnikar, 1994). In postmortem studies on subjects with MDD, there is a significant decrease in the number and density of astrocytes in the amygdala (Altshuler et al., 2010), prefrontal cortex (Miguel-Hidalgo et al., 2000), hippocampus (Gos et al., 2013; Cobb et al., 2016), and anterior cingulate cortex (Gittins and Harrison, 2011). On the contrary, in male rodents after NIA, when large doses of LPS are used, the number of astrocytes in the hippocampus increases both immediately after LPS administration (day 6; Zhao et al., 2022) and in adulthood (Berkiks et al., 2018a, 2019b). The activation of astrocytes in these studies is also accompanied by the emergence of a depressive state in adulthood (Berkiks et al., 2018a, 2019b; Zhao et al., 2022). The increase in the number of astrocytes affects the dentate gyrus to a lesser extent (Berkiks et al., 2019b; Liang et al., 2019). It is noteworthy that in females, after neonatal administration of LPS, the increase in the number of astrocytes and depressive behavior is observed less frequently: only in one of three studies. On the other hand, this phenomenon is more pronounced in CA1 and CA3 areas of the dorsal hippocampus, in contrast to males (Berkiks et al., 2019b; Table 2).

Thus, in contrast to MDD, after neonatal inflammation, the development of depression in rodents is accompanied by an increase in the activity/number of astrocytes, which is more pronounced in males than in females.

### Neurogenesis

Various types of stress, such as chronic social defeat, chronic immobilization, and early-life stress, reduce neurogenesis in the adult hippocampus (Anacker and Hen, 2017). Many studies have associated MDD with a decrease in adult hippocampal neurogenesis, including a decrease in the number of hippocampal granule neurons and neural stem/progenitor cells, a decrease in the volume of the hippocampus and of the dentate gyrus, and a decline of neurogenic-niche vascularization (Berger et al., 2020). Approaches involving various agents have been devised to assess neurogenesis. Administration of a thymidine analog (bromodeoxyuridine; BrdU), which is incorporated into newly synthesized DNA, enables the identification of newly divided cells (Kuhn et al., 1996). Endogenous marker doublecortin (DCX) helps to determine the number of immature neurons (Couillard-Despres et al., 2005). NeuN (neuronal nuclei) is commonly used as a biomarker of mature neurons, typically after they have downregulated DCX (Mullen et al., 1992). Consequently, a combination of immunoassays involving antibodies to different markers makes it possible to determine the ratio of mature to immature neurons and to quantitate neurogenesis.

After NIA, in the adult male hippocampus, the number of immature neurons (DCX^+^) does not change (Table 2; Dinel et al., 2014; Luo et al., 2021). The total number of proliferating cells (BrdU^+^) in the hippocampus does not change either (Liang et al., 2019; Luo et al., 2021). Nonetheless, postnatal LPS treatment leads to impairments of differentiation of late-stage progenitor cells into neurons (BrdU^+^/NeuN^+^ cells), thus causing accumulation of BrdU^+^/DCX^+^ cells in the group of the animals exhibiting depressive-like behavior (Liang et al., 2019). Furthermore, a second inflammatory stress in males showing depressive behavior after NIA induces a decrease in neurogenesis (a decline in the proliferation and number of immature neurons; Dinel et al., 2014). In a single study, in contrast to males, females with a history of NIA showed an elevated number of immature neurons in the dentate gyrus of the dorsal, but not ventral, hippocampus (Luo et al., 2021), consistently with less depressive behavior of the females.

Thus, after neonatal inflammation, the development of depression in males, but not females, is accompanied by a delay in the maturation of neurons during neurogenesis and a decrease in the magnitude of neurogenesis itself after additional stressors in adulthood.

## Molecular Signatures Detectable After NIA Associated with Depression

### Oxidative stress

Another cause of structural and functional brain anomalies in depression is oxidative stress. According to the oxidative stress hypothesis of depressive disorders, abnormalities in nitric oxide (NO) signaling enhance the production of reactive nitrogen and oxygen species and modulate functions of the main neurotransmitters of the CNS, HPA axis, and proinflammatory cytokines (Kudlow et al., 2016). Accumulation of lipid peroxidation products such as malondialdehyde also correlates with the severity of depressive symptoms (Mazereeuw et al., 2015). Normally, the formation of excessive amounts of reactive oxygen species (ROS) is neutralized by enzymes of the antioxidant system, such as superoxide dismutase (SOD) and catalase. In depression, however, one can detect both an increase in the activity of SOD and catalase (Vaváková et al., 2015)—in response to an increase in the number of free radicals—and a decrease in the activity of antioxidants (Gałecki et al., 2009).

After NIA, adult animals of both sexes have higher levels of nitric oxide (NO), lipid peroxidation, and the end product of lipid peroxidation (malondialdehyde; Berkiks et al., 2018a,b, 2019a,b; Benmhammed et al., 2019) and lower activity of the antioxidant enzyme SOD (Benmhammed et al., 2019) in the hippocampus and prefrontal cortex. Under additional stress, in males, but not females, myeloperoxidase (MPO) activity goes up in the hippocampus and prefrontal cortex (Custódio et al., 2018); this enzyme also mediates oxidative stress by stimulating the production of reactive nitrogen and oxygen species and by altering the polarization and inflammation-related signaling pathways in neutrophils and microglia.

Therefore, just as in MDD, after NIA, there is general suppression of the body’s antioxidant defenses and elevated oxidative stress accompanied by depressive behavior in adolescent and adult rodents.

Increased amounts of ROS induce cyclooxygenase 2 (COX-2) enzymatic activity, upregulating inflammatory prostaglandins and leading to mental illnesses (López and Ballaz, 2020). The administration of COX-2 inhibitors reverses depression-like behaviors by suppressing glial activation, ROS production, and neuronal apoptosis in rats (Gamble-George et al., 2016). Adult males, but not females, with a history of NIA have an elevated basal level of hypothalamic COX-2 (Boissé et al., 2005; Spencer et al., 2006; Kentner et al., 2010). Taken together, these signs correlate with the above-mentioned increase in the amount of reactive oxygen and nitrogen species in the brain.

Oxidative stress can induce a response of the HPA axis and of the immune system (cytokine production) to an inflammatory stimulus and may indirectly indicate persistent low-grade chronic inflammation after a neonatal challenge. Indirectly, this observation also confirms the decline of COX-2 activity in response to a second LPS challenge later in life in both sexes, thus pointing to the depletion of cell reserves for the production of this enzyme (Boissé et al., 2005; Spencer et al., 2006; Kentner et al., 2010).

### The HPA axis

One of the key functions in the regulation of depressive-like behavior is performed by stress hormones, the main of which is corticosterone in rodents. Stress hormones are often synergistically activated along with proinflammatory cytokines in response to the same stimulus (e.g., stress or inflammation). Therefore, an important part of the research into the consequences of NIA is the evaluation of the HPA axis in animals.

In adolescent animals, despite elevated basal levels of corticosterone, there is no increase in the production of this hormone in response to stress later in life (Tishkina et al., 2016; Yan and Kentner, 2017; Table 3), in agreement with the absence of patterns of depressive behavior. Adult animals (after postnatal day 60) with a history of NIA possess normal basal levels of corticosterone (Lucchina et al., 2010; Dinel et al., 2014; Tishkina et al., 2016).

**Table 3:** The influence of NIA on the magnitude of oxidative stress.

**Time after injection**	**PND**	**Dose LPS, mg/kg**	**Model**	**Sex**	**Depressive behavior**	**Hippocampus**	**PFC**	**Hypothalamus**	**Periphery**	**References**
**Adolescent**										
35 d	5+7	IP, 0.05 mg/kg	Swiss mice	m, f	↑	MPO*↑, m MPO* =, f	MPO* ↑, m MPO* =, f	MPO* ↑, m MPO* =, f		Custódio et al. (2018)
**Adult**										
70 d	5+7	IP, 0.05 mg/kg	Swiss mice	m, f	↑	MPO* ↑, m MPO* =, f	MPO* =, m, f	MPO* =, m, f		Custódio et al. (2018)
12–13 wk	14	IP, 0.25 mg/kg	Wistar rats	m	↑	NO ↑ MDA ↑				Berkiks et al. (2018a)
90 d	14	IP, 0.1 mg/kg	Sprague Dawley rats	m, f	=			COX-2 ↑ m COX-2* ↓, m COX-*2* = , f COX-2* ↓, f		Kentner et al. (2010)
3 mth	9	IP, 0.25 mg/kg	Wistar rats	m	↑		NO ↑ MDA ↑			Berkiks et al. (2018b)
90–97 d	1	IP, 0.25	Wistar rats	m, f	↑	NO ↑, m = f LPO ↑, m<f SOD ↓, m<f	NO ↑, m<f LPO ↑, m<f SOD ↓, m = f		Liver, spleen LPO ↑, m, f	Benmhammed et al. (2019)
11 mth	14	IP, 0.25 mg/kg	Wistar rats	m, f	m = f ↑	NOx ↑, m, f MDA ↑, m MDA = , f	NOx ↑, m, f MDA = , m MDA ↑, f			Berkiks et al. (2019a)

Comments: changes in depressive behavior were assessed if at least one of the special tests showed such behavior (Table 1). Abbreviations: m, male; f, female; IP, intraperitoneal administration; SC, subcutaneous administration; d, day; mth, month; wk, week; PND, postnatal day; ↑/↓, an increase or decrease in a parameter compared with a control, respectively; *25–30 min after the restraint or forced swim test/tail suspension test; SOD, superoxide dismutase; MDA, malondialdehyde (which is an end product of lipid peroxidation, is widely known as a good marker of oxidative stress); LPO, lipid peroxidation; NO, nitric oxide; NOx, nitrogen oxides; MPO, myeloperoxidase.

After the second stressor in adulthood (administration of an additional dose of LPS), corticosterone levels depend on the dose of neonatal administration of LPS. For instance, neonatal exposure to low doses of LPS (25 μg/kg) does not alter the response of this hormone to the LPS rechallenge: 2 h after LPS administration, the animals show an increased corticosterone level, just as controls do. On the other hand, neonatal exposure to higher doses of LPS (50 μg/kg) results in resistance and no upregulation of corticosterone after the adult LPS rechallenge (Lucchina et al., 2010; Bernardi et al., 2014). Immediately after behavioral tests, a stronger (than control) enhancement of corticosterone production takes place (Doosti et al., 2013; Majidi et al., 2016; Tishkina et al., 2016). Although in one study, corticosterone levels after behavioral testing did not differ between animals with a history of NIA and controls, only in the animals with a history of NIA did a comparison of hormone levels before and after behavioral testing revealed a significant ~2-fold increase in the corticosterone level (Tishkina et al., 2016).

Thus, in adult animals with a history of NIA, the response of the HPA axis to the second inflammatory stressor is attenuated, but acute physical stress induces a stronger hormonal response, which is accompanied by depressive behavior only in males.

### Cytokines

Greater activation of the HPA axis and high levels of oxidative stress has a direct impact on the production of proinflammatory cytokines. Animals with a history of NIA show no signs of inflammation in peripheral blood at baseline and after an adult LPS rechallenge; this finding may be due to the absence of a depressive phenotype in the animals in these studies (Table 4, Kentner et al., 2010; Bernardi et al., 2014; Yan and Kentner, 2017). Nonetheless, six out of the eight studies where brain tissues of depressed animals with a history of NIA have examined show persistence of low-grade inflammation judging by altered concentration of one or more proinflammatory cytokines (TNF, IL-1β, and IL-6; Table 4).

**Table 4:** Changes in corticosterone levels after neonatal inflammationn.

**Time after injection**	**PND**	**Dose LPS, mg/kg**	**Model**	**Depressive behavior**	**Sex**	**Plasma**	**References**
**Adolescent**							
30–35 d	3+5	SC, 0.05	Wistar rats	=	m	CORT ↑ CORT* =	Tishkina et al. (2016)
40 d	3+5	IP, 0.05	Sprague-Dawley rats	=	m, f	CORT#= m, f	Yan and Kentner (2017)
**Adult**							
60 d	3	IP, 0.025	F1 C57Bl/6xBalb	=	n.d.	CORT = CORT# =	Lucchina et al. (2010)
60 d	3	IP, 0.05	F1 C57Bl/6xBalb	m ↑ f =	n.d.	CORT = CORT# ↓	Lucchina et al. (2010)
60 d	3	IP, 0.025	F1 BalbxC57Bl/6	=	n.d.	CORT = CORT# =	Lucchina et al. (2010)
60 d	3	IP, 0.05	F1 BalbxC57Bl/6	=	n.d.	CORT = CORT# ↓	Lucchina et al. (2010)
60–70 d	2	IP, 0.05	Wistar rats	↓#	m, f	CORT# = m CORT#↓ f	Bernardi et al. (2014)
90 d	3+5	SC, 0.05	NMRI mice	↑	m	CORT* ↑	Majidi et al. (2016)
90 d	14	IP, 0.1	CD-1 mice	↑	m	CORT=	Dinel et al. (2014)
90 d	3+5	SC, 0.05	NMRI mice	↑	m, f	CORT* ↑ m, f	Doosti et al. (2013)
100–107 d	3+5	SC, 0.05	Wistar rats	↑	m	CORT = CORT* =	Tishkina et al. (2016)

Comments: changes in depressive behavior were assessed if at least one of the special tests showed such behavior (Table 1). Abbreviations: CORT, corticosterone; m, male; f, female; IP, intraperitoneal administration; SC, subcutaneous administration; d, day; mth, month; wk, week; PND, postnatal day; ^#^after an adult rechallenge with LPS; *25–30 min after the restraint or forced swim test/tail suspension test; ↑/↓, an increase or decrease in a parameter compared with the effect of stress in a control group, respectively; = , no difference from the effect of stress in the control group.

For example, at baseline, in adult males, but not females, manifesting depression-like behavior, a high level of the TNF protein persists for 3 months in the hippocampus and prefrontal cortex (Berkiks et al., 2018b, a) along with normal levels of IL-6 (Kvichansky et al., 2021; Table 5). Notably, mRNA levels of these cytokines show the opposite directions of alterations: *Tnf* mRNA expression is unchanged, whereas *Il-6* mRNA expression is elevated in the hippocampus of males (Kvichansky et al., 2021).

**Table 5:** The influence of neonatal administration of LPS on cytokines.

**Time after injection**	**PND**	**Dose LPS**	**Model**	**Sex**	**Depressive behavior**	**Hippocampus**	**PFC**	**Hypothalamus**	**Plasma**	**References**
**Adolescent**										
35 d	5+7	IP, 0.05 mg/kg	Swiss mice	m, f	↑	IL-4* ↑ m, f IL-6* ↓ m, f	IL-4* ↑ m, f IL-6* ↓ m, f	IL-4* ↑ m IL-4* = f IL-6* ↓ m, f		Custódio et al. (2018)
40 d	3+5	IP, 0.05 mg/kg	Sprague-Dawley rats	m, f	=				IL-10 n.d. m, f IL-10# = m, f	Yan and Kentner (2017)
**Adult**										
60–70 d	2	IP, 0.05 mg/kg	Wistar rats	m, f	↓#				TNF#= m = f IL-1β#= m = f	Bernardi et al. (2014)
70 d	5+7	IP, 0.05 mg/kg	Swiss mice	m, f	↑	IL-4* ↑ m, f IL-6* ↓ m, f IFNγ* ↑ m, f	IL-4* ↑ m IL-4* = f IL-6* ↓ m, f	IL-4* ↑ m, f IL-6* ↓ m, f		Custódio et al. (2018)
90 d	14	IP, 0.1 mg/kg	Sprague-Dawley rats	m, f	=				IL-6 = m, f IL-6# ↓ m, f	Kentner et al. (2010)
3 mth	3+5	SC, 0.05 mg/kg	Wistar rats	m, f	↑	IL-6 = m, f IL-6* = m, f*Il-6 ↑ m Il-6 = f* Il-6* = m, f*Tnf =* m, f Tnf* = m, f Il1b = , m, f Il1b* =, m, f Il-10 not detectable				Kvichansky et al. (2021)
3 mth	9	IP, 0.25 mg/kg	Wistar rats	m	↑		TNF ↑			Berkiks et al. (2018b)
3 mth	14	IP, 0.25 mg/kg	Wistar rats	m, f	↑	TNF* =, m<f				Berkiks et al. (2019b)
12–13 wk	14	IP, 0.25 mg/kg	Wistar rats	m	↑	TNF ↑				Berkiks et al. (2018a)
100 d	3+5	IP, 0.05 mg/kg	NMRI mice	m, f	↑	TNF* ↑ IL-1β* ↑				Majidi et al. (2016)
11 mth	14	IP, 0.25 mg/kg	Wistar rats	m, f	m = f ↑	TNF* = m TNF* = f	TNF* = m TNF* ↑ f			Berkiks et al. (2019a)

Comments: changes in depressive behavior were assessed if at least one of the special tests showed such behavior (Table 1). Abbreviations: m, male; f, female; IP, intraperitoneal administration; SC, subcutaneous administration; d, day; mth, month; wk, week; PND, postnatal day; DH, dorsal hippocampus; VH, ventral hippocampus; ^#^after an adult rechallenge with LPS at 0.1 mg/kg; *25–30 min after the restraint or forced swim test; ↑/↓, an increase or decrease in a parameter compared with the effect of stress in a control group, respectively; = , no difference from the effect of stress in the control group.

In animals with a history of NIA who show depressive behavior, mRNA levels of *Il1b*, *Il6*, and *Tnf* do not change in the hippocampus after behavioral tests (Kvichansky et al., 2021) as compared to a control but decline relative to the baseline of the animals with a history of NIA. mRNA expression does not necessarily reflect protein expression on the cell surface. Indeed, under severe stress, such as the restraint test, there is upregulation of IL-1β and TNF in the hippocampus and prefrontal cortex (Majidi et al., 2016). On the other hand, behavioral tests alone do not alter the amounts of these cytokines (Berkiks et al., 2019a,b). Of note, after behavioral tests, the level of IL-6 in the hippocampus and prefrontal cortex decreases both relative to baseline in females with a history of NIA (Kvichansky et al., 2021) and in comparison with the control group, regardless of sex and age (Custódio et al., 2018). Because IL-6 participates in the preservation of neurogenesis, in neuronal differentiation, and in the protection of neurons from damage, a decrease in the amount of this cytokine can have negative consequences for the homeostasis of the nervous system, by preventing tissue repair after inflammation (García-Juárez and Camacho-Morales, 2022).

Accordingly, the absence of any changes in levels of cytokines in blood plasma either at baseline or after stress, together with the imbalance of cytokines in brain tissues, indicates long-term persistence of local inflammation, which is accompanied by the development of depressive behavior too. It should also be noted that in adult females, the basal level of TNF, just as the magnitude of microglial activation (Berkiks et al., 2019b; Kvichansky et al., 2021) and the level of oxidative stress (Benmhammed et al., 2019), is higher than that in males (Berkiks et al., 2019b). Nonetheless, after 11 months, it is females with depression-like behavior who show an increased concentration of TNF in the prefrontal cortex (Berkiks et al., 2019a), whereas in males, this concentration does not differ from the level of control females and between control males and males with a history of NIA. It can be theorized that the elevated basal levels of these parameters in females in the early period of life serve as a protective factor against the adverse effects of inflammation and prevent the development of depressive behavior.

## Discussion and Perspectives

Our analysis of the literature suggests that owing to persisting molecular changes in brain tissues, early-life neonatal inflammation is a risk factor for depression-like behavior in adult males only when they face aversive/difficult life situations. Elevated oxidative stress raises glial activation and stimulates proinflammatory signaling pathways, thereby possibly prolonging/perpetuating inflammation and contributing to the abnormalities of brain functions and of neuronal signaling that are seen in depression (Bhatt et al., 2020). A cytokine imbalance and altered reactivity of the HPA axis to a second stressor—along with normal basal levels of corticosterone, TNF, and IL-1β—are consistent with the “two-hit theory” of depression development, according to which exposure to environmental stress at an early age enhances behavioral and hormonal responses to an additional stressor in adolescence and adulthood (Calcia et al., 2016; Reshetnikov et al., 2021). Furthermore, the combined effects of reactive oxygen and nitrogen species and of inflammatory cytokines reduce the availability of tetrahydrobiopterin (BH4): a key enzyme cofactor for the synthesis of all monoamines (Neurauter et al., 2008). It is also known that upregulation of IL-1β and TNF stimulates the function of serotonin reuptake transporters through IL-1R and the p38 MAPK pathway, thereby lowering the synaptic availability of serotonin and causing a manifestation of depressive behavior in laboratory animals (Zhu et al., 2010).

Back in 1993, T.B. Herbert and S. Cohen reported an association between the development of clinical depression and alterations in cellular immunity (Herbert and Cohen, 1993). In case-control studies on MDD, genes connected with a response to infection and with innate immunity have been shown to be overexpressed (Leday et al., 2018; Wittenberg et al., 2020), in agreement with data indicating activation of innate immunity and enhanced signaling of proinflammatory cytokines (Dantzer et al., 2008; Miller et al., 2009). At the same time, genes related to T-cell function and adaptive immunity are underexpressed (Leday et al., 2018), also consistently with previous findings pointing to a relative decrease in natural killer cytotoxicity and in lymphocyte proliferation under the action of mitogens *in vitro* (Zorrilla et al., 2001) and with recent data suggesting that natural killer and T-cell deficiency is accompanied by monocyte inflammatory activation (Grosse et al., 2016; Snijders et al., 2016).

The described molecular alterations in depressed animals with a history of NIA have pathogenetic patterns similar to those in patients with MDD and in depressive-like behavior after NIA (Table 6). This observation suggests that early-life neonatal infection causes long-term alterations of the already characterized pathogenic changes in MDD. For instance, some of the genes overexpressed in MDD are genes participating in the response to LPS (Leday et al., 2018), consistently with overexpression of TLR4 in the prefrontal cortex in depressed subjects and suicide victims (Pandey et al., 2019). This phenomenon may also be the reason for the over-reaction of cytokines during inflammation in adults after NIA.

**Table 6:** A comparison of molecular features between MDD and depression after NIA.

**Features**	**MDD**	**Depressive-live behavior after NIC**
Cytokines	TNF↑, IL-1β↑, IL-6↑, IL-17↑	TNF↑, IL-1β↑, IL-6↓/↑, IL-17?
HPA axis	CORT↑*, GR-resistant	CORT↑*
Glia reaction, neurogenesis	Microglia activation, density, engulfment ↑, astrocyte number, and density ↓; neurogenesis ↓	Microglia activation, density, engulfment ↑, astrocytes ↑, neurogenesis ↓
Oxidative stress	NO↓, LPO↑, MDA↑, MPO, SOD↑/↓, COX-2	NO↑, LPO↑, MDA↑, MPO↑, SOD↓, COX-2↓
Immune system	Th-17↑, Th-1↑, Treg↓	Th-17?, Th-1?, Treg?

Abbreviations: CORT, corticosterone; COX-2, cyclooxygenase-2; GR, glucocorticoid receptor; HPA, hypothalamic–pituitary–adrenal axis; IL, interleukin; LPO, lipid peroxidation; MDA, malondialdehyde (which is an end product of lipid peroxidation, is widely known as a good marker of oxidative stress); MDD, major depressive disorders; MPO, myeloperoxidase; Treg, regulatory T cells; NIC, neonatal immune challenge; NO, nitric oxide; SOD, superoxide dismutase; Th, T-helper lymphocyte; TNF, tumor necrosis factor; ↑/↓, an increase or decrease in a parameter compared with a control, respectively; *25–30 min after aversive conditions (the forced swim test, tail suspension test, or restraint test).

Aside from hypersensitivity to inflammatory stimuli, anomalies in the regulation of immune responses are seen in depression. It has been shown that the depletion of regulatory T cells (CD4^+^ CD25^+^ Tregs) *in vivo* can lead to anxious and depressive behavior (Kim et al., 2012) *via* increased inflammation (Grosse et al., 2016; Snijders et al., 2016). A case-control study indicates that after effective antidepressant therapy, the number of regulatory T cells in the blood goes up and the number of T helper 17 (Th17) cells diminishes (Jahangard and Behzad, 2020). Recent research also indicates that CD4^+^ CD25^+^ regulatory T cells can inhibit inflammatory responses in depressed patients (Ellul et al., 2018). A transfer of off-spring antigen–specific regulatory T cells from the dams that had toxoplasmosis during pregnancy reverses pups’ behavioral abnormalities, thus implying the therapeutic potential of adoptive regulatory-T-cell transfer in neuropsychiatric disorders associated with immune alterations (Xu et al., 2021). This is a rare piece of direct evidence linking regulatory T cells and behavior. Furthermore, a decrease in plasma serotonin concentration correlates with the downregulation of the 5-HT1a receptor on the surface, but not in the cytoplasm, of regulatory T cells in depressed patients (Li et al., 2010). Because serotonin takes part in the activation of lymphocyte proliferation through the 5-HT1a receptor (Aune et al., 1993), the decrease in the concentration of this monoamine in the blood reduces the proliferation and the number of regulatory T cells.

The microbiome also plays a considerable role in the development and persistence of MDD (Jiang et al., 2015), mainly owing to an imbalance between Th17 cells and regulatory T cells toward the former (Westfall et al., 2021a,b). IL-17, produced by RORγt^+^ Th17 cells, contributes to the development of depression, especially its treatment-resistant subtypes (Nothdurfter et al., 2021). The literature shows that the number of Th17 cells in gut-associated lymphoid tissue increases in response to stress and correlates with depression-like behavior in mice (Westfall et al., 2021b). Besides, administered Th17 lymphocytes have been found to cause depression-like behavior in mice (Beurel et al., 2013).

Because of the similarity of molecular alterations that occur during MDD and after NIA as well as the already shown influence of the microbiota and Th17 or regulatory T cells on the development and course of depression, a question arises: what effect does neonatal inflammation have on the emerging microbiota of pups and on the establishment of a balance between Th17 cells and regulatory T cells? It is known that regulatory T cells arise in the thymus and begin to migrate to peripheral organs and tissues, primarily to the skin and intestinal walls, at early stages of embryogenesis (Scharschmidt et al., 2015, 2017) and are necessary to maintain tolerance to one’s own emerging microbiota (Mold et al., 2008; Lathrop et al., 2011; Ohnmacht et al., 2015; Westfall et al., 2021b). In newborn mice, it has been shown that the primary response to an antigen induces Th1 and Th2 immune responses, with equal production of interferon gamma (IFN-γ) and IL-4 (Mold et al., 2008). During an antigen rechallenge, rapid production of IL-4 occurs, which causes apoptosis of neonatal Th1 cells through the formation of heteroreceptor IL-4Rα/IL-13Rα1, which together with regulatory T cells takes part in the control over peripheral tolerance and restrains autoimmunity in adults (Barik et al., 2017). This observation further confirms the initial tolerogenic type of the immune system in newborns.

Overall, the phenotypic diversity of regulatory T cells allows them to access various tissues, including brain tissue, where they implement immune homeostasis (Tanoue et al., 2016; Khantakova et al., 2022b). In the CNS, regulatory T cells play a major part in the regulation of inflammation. In the rat brain, regulatory T cells constitute ~15% of CD4^+^ T lymphocytes (Xie et al., 2015). Notably, brain astrocytes may perform a regulatory function in the control of cerebral regulatory T cells by promoting the expression of Foxp3 *via* the IL-2–STAT5 pathway (Xie et al., 2015). Neurons also contribute to the differentiation of regulatory T cells, *via* B7 family proteins and the TGF-β1 pathway (Liu et al., 2006). Stressors during the critical period of immune-system formation and migration of regulatory T cells can have long-term and unpredictable consequences, by reducing their functional activity and migration potential.

Of note, the sex- and age-specific outcomes that we found were somewhat unexpected. The changes we noted in adult males (but not in females or adolescents regardless of gender) make us wonder which molecular mechanisms determine the timing of the manifestation of delayed effects of NIA and its gender specificity. Human studies show that childhood infectious diseases increase the risk of psychotic illness in adulthood regardless of gender (Dalman et al., 2008; Khandaker et al., 2012). Nonetheless, a recent population-based longitudinal birth cohort study (Chaplin et al., 2020) revealed that early-childhood infections are associated with the risk of depressive symptoms in adolescence (age 12–17 years) but not in early adulthood (age 18–19 years). The inconsistency of the results about humans may be due to the population’s genetic background or to the socioeconomic characteristics of the subjects and to the assessment methods. After interpreting the data, we can hypothesize that behavioral patterns in mice during adolescence make it difficult to detect depression-like behavior in the tail suspension test and forced swim test. It is known that adolescent rodents are less anxious than juvenile or adult rodents and are more prone to risk-taking, impulsive behavior, and novelty-seeking (Macrì et al., 2002; Stansfield and Kirstein, 2006; Arrant et al., 2013). It is possible that these features of the behavior of mice during adolescence ensure the best stress-coping behavior under aversive conditions, making differences in the manifestations of depressive-like behavior between groups insignificant. On the other hand, the scarcity of studies on adolescent and female rodents in this field may also be one of the reasons for the lack of pronounced differences. Accordingly, in the future, to assess changes in rodents during adolescence, it will be necessary to increase the number of studies and to develop specific tests for depressive-like behavior in adolescent animals.

In conclusion, our analysis uncovered delayed structural and molecular patterns linked with the development of depression-like behavior in rodents after neonatal inflammation. Due to the similarity of the identified changes with data from MDD patients, further studies should address the influence of neonatal inflammation on the differentiation and functioning of the immune system’s regulatory mechanisms as well as the search for possible ways to correct the identified disturbances.

## Limitations

The analysis of the impact of delayed consequences of induced neonatal inflammation in rodents has several limitations regarding the interpretation of data on neuroinflammation and depression-like behavior. The first set of limitations is related to the heterogeneity of experimental data (e.g., the lineage, mimetic administration protocol, age of analysis of behavior and molecular changes, the type of test for behavior assessment, the brain structure chosen for analysis, the extraction method, coordinates, and assessment techniques). The heterogeneous design of experiments means that we can analyze only the most pronounced changes that are common among most models but cannot examine the less pronounced effects of a particular model. Apparently, the heterogeneity of the experimental design may be the reason for the inconsistent results about the influence of NIA on both behavioral parameters and structural and molecular characteristics of neuroinflammation.

The second limitation of the animal model for evaluating the effect of NIA on depression-like behavior is related to the limited range of detectable behavior in rodents, in contrast to humans. The most common tests currently available for assessing depressive-like behavior (the forced swim test, tail suspension test, or sucrose preference test) are able to quantify only a limited range of behavioral traits associated with depression. Such symptoms as low mood and low self-esteem, worthlessness, a feeling of guilt, impaired concentration, and suicidal behavior cannot be assayed in animals but are common manifestations of depression in humans. The commonly used tests reflect specific clinical symptoms, typically associated with a lack of motivation, but do not permit assessment of deeper anomalies of mood and behavior. The forced swim test and tail suspension test involve well-pronounced aversive conditions for rodents, and according to some authors, evaluate stress-coping strategies rather than depressive-like behavior (Molendijk and de Kloet, 2015, 2019; de Kloet and Molendijk, 2016). Nonetheless, due to the simplicity and affordability as well as the complexity of alternative techniques, these tests continue to be popular in scientific research.

Finally, the third limitation is brain morphofunctional differences between rodents and humans. These differences are seen both in the neonatal period—when the stage of development of the brain in rats and mice corresponds to the stage of development of the third trimester of pregnancy in humans (Lupien et al., 2009)—and in adulthood because it is not yet possible to unambiguously match various regions of the cerebral cortex between rodents and humans. This may be the reason for the inconsistency among results of comparisons of transcriptional signatures between various mouse models of depression and postmortem data from patients with MDD (Scarpa et al., 2020; Reshetnikov et al., 2022).

Despite the limitations associated with the use of animal models of NIA, our analysis points to a close relationship between inflammation at an early age and the development of a depressive state in adults when exposed to aversive environmental conditions. This conclusion also confirms the similarity of the alterations detectable after NIA at the cellular and molecular levels with the pathological changes that proceed during depression.

## Author Contributions

JK and EA searched databases independently to select the articles. JK wrote the manuscript. EA prepared the tables. NB and VR planned and supervised the whole project. All authors contributed to the article and approved the submitted version.

## Footnotes

^1^ https://www.nimh.nih.gov/health/statistics/major-depression
